# The Pathology and Treatment of Venereal Diseases

**Published:** 1883-11

**Authors:** 


**The Pathology and Treatment of Venereal Diseases.** By FREEMAN J. BUMSTEAD, M. D., LL. D., late Professor of Venereal Diseases at the College of Physicians and Surgeons, New York, etc., and ROBERT W. TAYLOR, A. M., M. D., Professor of Venereal and Skin Diseases in the University of Vermont, President of the American Dermatological Association, etc. Fifth edition, revised and rewritten, with many additions by Dr. Taylor, with 139 wood-cuts and thirteen chromo-lithographic figures. Philadelphia: Henry C. Lea's Son & Co. 1883.

In answer to the numerous inquiries which come to us from students and practitioners as to the best work on venereal diseases, we have said: "If you can buy but one, get Bumstead." Our good opinion of the book is strengthened by a careful review of this last edition. Dr. Taylor has shown his ability and his intention to keep the book in the front rank with the many admirable works on venereal diseases now presented to the profession. Much new matter has been added relating to therapeutics. The subjects of the inoculation of animals with syphilis and the bacillus origin of the disease are fully discussed and a chapter on syphilis and marriage has been appended. The chromo-lithographic plates are a useful addition. The work is on a level with our present knowledge in all particulars. It is eminently practical, well written, full of excellent counsel, and worthy of the same confidence and appreciation which the previous four editions have so markedly enjoyed.

